# Habitat Correlates of the Red Panda in the Temperate Forests of Bhutan

**DOI:** 10.1371/journal.pone.0026483

**Published:** 2011-10-19

**Authors:** Sangay Dorji, Karl Vernes, Rajanathan Rajaratnam

**Affiliations:** 1 Jigme Dorji National Park, Department of Forests and Park Services, Ministry of Agriculture and Forests, Bhutan; 2 Ecosystem Management, University of New England, Armidale, New South Wales, Australia; 3 Geography and Planning, University of New England, Armidale, New South Wales, Australia; University of Otago, New Zealand

## Abstract

Anthropogenic activities and associated global climate change are threatening the biodiversity in the Himalayas against a backdrop of poor knowledge of the region's threatened species. The red panda (*Ailurus fulgens*) is a threatened mammal confined to the eastern Himalayas, and because of Bhutan's central location in the distributional range of red pandas, its forests are integral to the long-term viability of wild populations. Detailed habitat requirements of the red panda are largely speculative, and there is virtually no ecological information available on this species in Bhutan. Between 2007 and 2009, we established 615 presence/absence plots in a systematic sampling of resident habitat types within Jigme Dorji and Thrumshingla National Parks, Bhutan, to investigate broad and fine-scale red panda habitat associations. Additional locality records of red pandas were obtained from interviewing 664 park residents. Red pandas were generally confined to cool broadleaf and conifer forests from 2,110–4,389 m above sea level (asl), with the majority of records between 2,400–3,700 m asl on south and east-facing slopes. At a finer scale, multivariate analysis revealed that red pandas were strongly associated with old growth Bhutan Fir (*Abies densa*) forest dominated by a dense cover of *Yushania* and *Arundanaria* bamboo with a high density of fallen logs and tree stumps at ground level; a high density of trees, dead snags, and rhododendron shrubs in the mid-storey; and locations that were close to water. Because Bhutan's temperate forests that encompass prime red panda habitat are also integral to human subsistence and socio-economic development, there exists an inadvertent conflict between the needs of people and red pandas. As such, careful sustainable management of Bhutan's temperate forests is necessary if a balance is to be met between the socioeconomic needs of people and the conservation goals for red pandas.

## Introduction

The eastern Himalayas, which stretch from central Nepal in the west to Myanmar in the east, taking in southeast Tibet in China, north-east India, and Bhutan, encompasses a biologically diverse region of global importance. The eastern Himalayas contain a high degree of plant endemism [1], two Endemic Bird Areas [2], and several centres for plant diversity [3]. As such, the region is recognised as a global biodiversity hotspot [4] and a priority for conservation as a Global 200 ecoregion [5]. Several high profile mammals of global conservation significance occur in the region, including tiger *Panthera tigris*, snow leopard *Panthera uncia*, takin *Budorcas taxicolor*, Himalayan black bear *Ursus thibetanus* and red panda *Ailurus fulgens* [3]. However, biodiversity values of the eastern Himalayan forests are being eroded by an expanding human population that relies on these forests for livestock grazing, timber extraction, and the harvesting of fuel-wood, bamboo, fodder and fertilizer. Furthermore, the Greater Himalayan region is particularly sensitive to the impacts of global warming, with increases in temperature already occurring at three times the global average [6], with modeling predicting significant changes to the distribution, extent, and composition of the region's ecosystems [6]. Current knowledge of the ecology and distribution of most threatened species across the eastern Himalayas is generally poor [7], creating great uncertainty about the magnitude and trajectory of change that may occur in abundance and distribution of individual species [6]. As such, there is a pressing need to understand the ecological requirements of the region's threatened biota, particularly umbrella, indicator and flagship species, if the balance between human socioeconomic development and nature conservation is to be achieved.

The red panda is endemic to the eastern Himalayas, and with the exception of a small isolated tropical forest population in India, the species is confined to temperate conifer forest and adjacent broadleaf forest [8] where it specialises on a diet of bamboo [9], [10]. Its distribution ranges from western Nepal into India, Bhutan, and northern Myanmar through to the Minshan Mountains and upper Min Valley of Sichuan Province in south-central China [7], [8], [11]. Because of a reliance on mature conifer forest with a complex understorey, coupled with a broad distribution across the eastern Himalayas, the red panda has been proposed as a suitable indicator species for monitoring the integrity of the Eastern Himalayan Broadleaf and Conifer Eco-region [12]. Red pandas are charismatic mammals making them an ideal flagship species for harnessing public support for prudent natural resource management in the Eastern Himalayas [7], [13]. However, the red panda is vulnerable to extinction through habitat loss and fragmentation, which restricts the availability of mature den trees and prolific bamboo undergrowth [8], [11], [12], [14], [15], [16]. Because of Bhutan's central location in the distributional range of the red panda, its temperate forests are crucial to red panda population viability in the eastern Himalayas.

The few detailed studies on red panda ecology throughout its range suggest that bamboo cover and height, canopy cover, and proximty to water are important structural attributes [13], [15], [17]. However, other habitat-related studies on the red panda have only broadly examined its habitat requirements [12], [18], [19], [20], with most published information on the species limited to reviews on status and conservation [7], [8], [12], [19]. Futhermore, because published information on the red panda is restricted to studies conducted in India, China and Nepal, there is a pressing need to fill the gap in information on red pandas in Bhutan.

Here, we present a thorough study on habitat requirements of the red panda within the temperate forests of Bhutan. We investigated broad-scale associations with forest type along an altitudinal gradient, and fine-scale associations with specific habitat attributes in forest types confirmed to contain red pandas. This study is the most comprehensive to date on habitat correlates of the red panda, and fills an important gap in understanding its habitat requirements while adding to the paucity of ecological data on this globally threatened species.

## Results

### Presence-absence surveys

**Figure 1:**
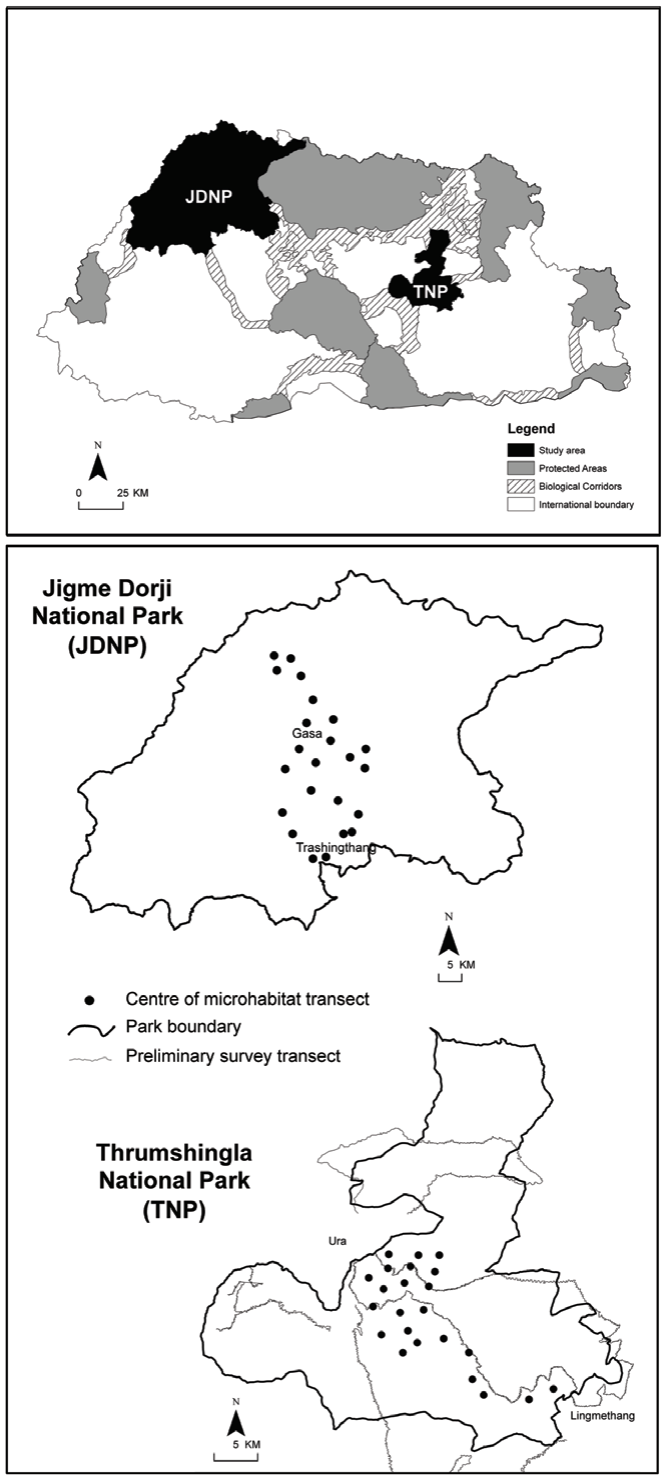
Map of the Kingdom of Bhutan, showing the locations of study area within Jigme Dorji and Thrumshingla National Parks.

**Figure 2:**
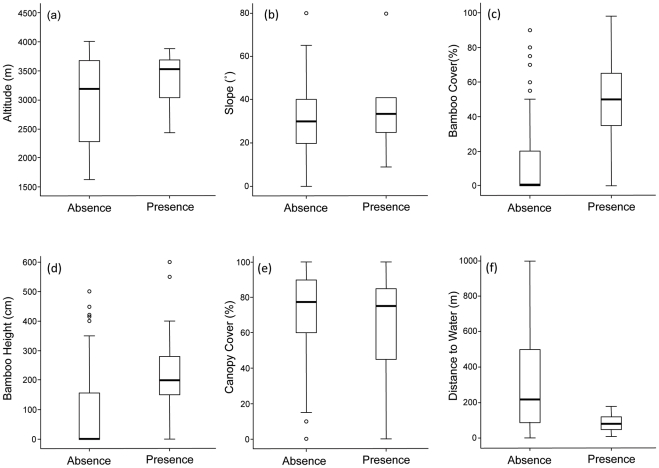
Box and whiskers plots comparing median (dark line), range (box), lower and upper quartile (whiskers) and outliers (open circles) of habitat variables in ‘Presence’ (plots where red panda were detected) and ‘Absence’ plots for (a) altitude; (b) slope; (c) bamboo cover (d) bamboo height; (e) canopy cover; and (f) distance to water.

From November 2007 to April 2008, we conducted preliminary surveys for red pandas in Thrumshingla National Park, Bhutan, to obtain base-line information on red panda occurrence in resident forest types and at various elevations. We then used these surveys to establish sampling plots for subsequent evaluation of micro-habitat association in Thrumshingla and Jigme Dorji National Park between December 2007 and December 2009 (see Fig. 1). Red pandas were present in 1.5% (n = 5) of the 339 random survey plots in Thrumshingla. Detections were in cool broadleaf forest (0. 3%; n = 1) and conifer forest (1.2%; n = 4) ranging from 2980–3685 m asl (Fig. 2). We did not detect red pandas in subtropical forest, warm broadleaf forest and alpine meadows. Presence of red pandas in cool broadleaf forest and conifer forest was supported by questionnaire surveys conducted on park residents. Out of 324 questionnaires, 11.7% (n = 38) of respondents could positively identify a red panda and had sighted an individual, with 2.6% (n = 1) and 97.4% (n = 37) of the sightings made in cool broadleaf forest and conifer forest, respectively. Questionnaire surveys in Jigme Dorji revealed similar habitat affiliations. Out of 340 questionnaires, 15.9% (n = 54) of respondents could positively identify a red panda and had sighted an individual, with 42.6% (n = 23) and 57.4% (n = 31) of the sightings made in cool broadleaf forest and conifer forest, respectively.

### Microhabitat association plots

Through systematic stratified sampling along 46 survey transects, we established 132 and 144 microhabitat association plots in Jigme Dorji and Thrumshingla, respectively, with 7.6% (Jigme Dorji: n = 10) and 8.3% (Thrumshingla: n = 12) being animal-presence plots (where red panda presence had been detected) and the remainder being animal-absence plots (where red panda presence was not detected). A number of landscape and structural habitat attributes were measured in each plot (Table 1). Red pandas were only detected in cool broadleaf forest and conifer forest plots from 2110–4389 m asl, with conifer forest containing a higher percentage of animal-presence plots (Jigme Dorji: conifer = 70% (n = 7), cool broadleaf = 30% (n = 3); Thrumshingla: conifer = 83.3% (n = 10), cool broadleaf = 16.7% (n = 2). Opportunistic animal-presence plots (n = 36) also revealed a higher percentage of these plots in conifer versus cool broadleaf forests (Jigme Dorji: conifer = 68.4% (n = 13), cool broadleaf = 31.6% (n = 6); Thrumshingla: conifer = 82.4% (n = 14), cool broadleaf = 17.6% (n = 3). Overall red panda occurrence within microhabitat association plots was significantly greater in conifer compared to cool broadleaf forests (*x* ^2^ = 3.92; p = 0.047). Of the 17 animal-presence plots in conifer forest, 76.5% (n = 13) were specifically associated with fir forest from 3000–3880 m asl.

**Table 1:** The habitat variables measured in each 20×20-m animal presence and animal absence plot in Jigme Dorji and Thrumshingla National Parks, Bhutan, and the unit of measurement and equipment used to measure each variable.

Variable Name	Unit of measurement	Equipment
Altitude above sea level	m	Altimeter
Slope	°	Suunto Clinometer
Aspect	E, W, N, S, SE, SW, NE, NW	Suunto Compass
Geographical location	UTM	GPS e-trex vista
Tree taxa	Total number	
Tree diameter	cm (DBH)	Diameter tape
Tree Height	m	Clinometer
Shrub taxa	Total number	
Logs	Total number	
Snag trees	Total number	
Bamboo cover	%	Visually estimated to 5%
Bamboo height	cm	Measuring tape
Canopy cover	%	Bamboo tube with cross hair
Distance to water	m	Hip-chain
Distance to road	km	Estimated to nearest km
Distance to settlement	km	Estimated to nearest km

### Site occupancy

Site occupancy modelling (based on animal-presence versus animal-absence plots) using PRESENCE predicted red pandas to occur in 32% (290 km^2^) of Thrumshingla's total area, and 26% (1130 km^2^) of Jigme Dorji's total area. Red panda detection probability was higher in Jigme Dorji (0.45) compared to Thrumshingla (0.35), suggesting that it was more readily detected in Jigme Dorji, despite the lower predicted occupancy. There was no significant difference between the two parks in the number of red panda detections (Fisher exact test, p = 0.812) so we pooled data for all subsequent analyses.

### Habitat and Landscape Attributes

**Figure 3:**
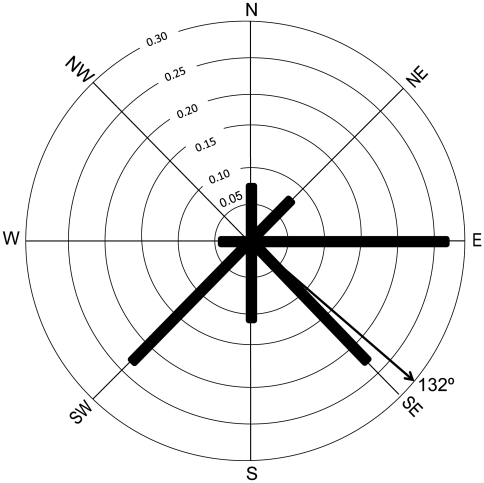
A relative frequency circular histogram of red panda occurrence according to aspect of plots. Heavy lines indicate the frequency occurrence of aspects of ‘animal presence’ plots (plots where red panda were detected), with the black arrow showing the mean aspect of these plots.

Animal-presence plots were characterised by a mean (± s.d.) altitude of 3342±463 m (Fig. 2a) mean (± s.d.) slope of 34±16° (Fig. 2b), mean (± s.d.) bamboo cover of 45±24% (Fig. 2c), mean (± s.d.) bamboo height of 230±139 cm (Fig. 2d) and mean (± s.d.) canopy cover of 66±29% (Fig. 2e). More than 70% of red panda droppings were encountered within 100 m of a water source, while 95% were within 150 m of a water source (overall mean ± s.d. = 80±46 m, Fig. 2f). Red pandas also displayed a significant association with easterly and southerly slopes, compared to northerly or westerly slopes (*Χ* ^2^ = 21, df = 6; p = 0.02; Fig. 3).

Bamboo cover, bamboo height, distance to water and number of large-stem fir, mid-stem fir and fruiting *Sorbus* trees differed significantly between animal-presence (n = 17) and animal-absence plots (n = 63) in conifer forest (Table 2). Altitude, the number of fruiting *Sorbus* trees, bamboo cover and bamboo height differed significantly between animal-presence (n = 5) and animal-absence plots (n = 58) in cool broadleaf forest (Table 2).

**Table 2:** Statistical results for Mann Whitney Z-test comparisons between the animal presence and animal absence plots in conifer and broadleaf forests of Jigme Dorji and Thrumshingla National Park, Bhutan.

Variable	Conifer Forest				Cool broadleaf Forest			
	Mean	s	Z	p	Mean	s	Z	p
Altitude (m)	3583	255	−0.982	0.326	2279	486	−2.120	**0.034** *
Mature trees	0.31	0.34	−0.847	0.397	0.48	0.37	−0.724	0.469
Mid-storey trees	0.36	0.37	−0.306	0.759	0.29	0.38	−0.214	0.830
Under-growth	0.57	0.61	−0.345	0.730	0.77	0.63	−0.311	0.756
Slope (°)	33.13	18.19	−0.230	0.818	28.94	12.65	−1.955	0.051
Mid-storey fir	1.78	2.80	−2.344	**0.019** *	0.00	0.00	-	-
Mature fir	4.69	4.69	−1.942	0.052	0.00	0.00	-	-
*Rhododendron*	3.22	4.61	−0.816	0.414	0.97	2.73	−1.944	0.052
Fruiting *Sorbus*	0.44	1.08	−6.467	**<0.001** *	0.05	0.28	−4.814	**<0.001** *
Snag trees	4.06	5.41	−0.775	0.439	1.15	1.51	−0.055	0.956
Log	4.10	4.28	−0.417	0.676	0.65	1.19	−1.516	0.130
Bamboo cover (%)	14.79	24.04	−5.212	**<0.001** *	22.55	27.47	−3.167	**0.002** *
Bamboo height (cm)	72.23	108.41	−5.100	**<0.001** *	162.50	173.32	−2.282	**0.023** *
Canopy cover (%)	71.19	23.40	−1.068	0.285	70.97	26.53	−0.480	0.631
Distance to water (m)	300.98	318.33	−3.094	**0.002** *	247.60	240.46	−1.942	0.052

*P<0.05.

### Multivariate Habitat Analyses

**Figure 4:**
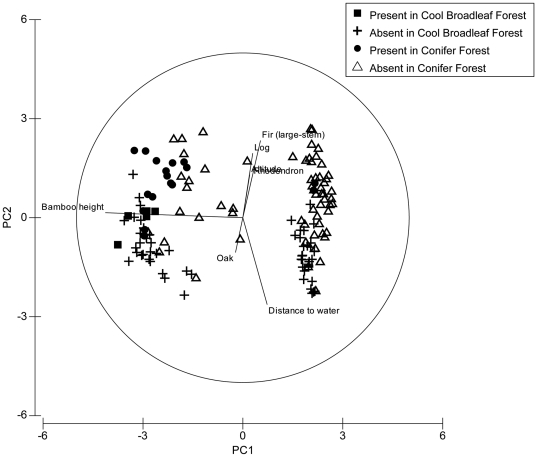
Principle Component Analysis showing relationship between ‘animal presence’ (plots with red panda evidence) and ‘animal absence’ plots in conifer and cool broadleaf forests plots, in Jigme Dorji and Thrumshingla National Parks, Bhutan.

To examine the importance of tree taxa to red pandas irrespective of habitat type, we generated an Important Value Index (IVI) of tree species in each plot irrespective of forest type. This analysis ranked Bhutan Fir (92.1), rhododendron (36.8), oak (22.4) and *Castanopsis* (16.4) as the four most important tree taxa in our study areas. A Principle Component Analysis (PCA) on pooled data between cool broadleaf forest and conifer forest narrowed habitat variables into three principle components that explained 71.4% of the total variance. PC1 explained 45.9% of the total variation, which was attributed to bamboo cover and bamboo height, with both bamboo cover and height decreasing along this axis (Table 3, Fig. 4). PC2 explained 13.8% of total variance and was attributed to decreasing values in altitude, mid-stem fir, large-stem fir, rhododendrons, tree stumps and snags, and logs but with an increase in distance to water and number of oak trees along the axis. This axis therefore largely distinguishes cool broadleaf forest plots at the positive end of the axis, and conifer forest plots at the negative end (Table 3, Fig. 4). PC3 explained 11.7% of the total variation with distance to water, large stem fir, rhododendrons, logs and bamboo height increasing along the axis (Table 3, Fig. 4).

**Table 3:** Principle Component scores^1^ for PCA of conifer and cool broadleaf forest plots in Jigme Dorji and Thrumshingla National Parks, Bhutan.

Variable	PC1	PC2	PC3
Altitude	0.042	**−0.271**	0.127
Slope	−0.013	−0.029	0.043
Distance to water	0.145	**0.449**	**0.865**
Distance to settlement	0.009	−0.152	0.051
Distance to road	0.026	0.006	−0.056
Large-stem trees	−0.014	0.069	0.046
Mid-stem trees	0.030	−0.062	0.002
Shrubs	0.021	0.104	0.045
Oak	−0.045	**0.210**	−0.047
*Castanopsis*	−0.016	0.126	−0.027
Mid-stem fir	0.066	**−0.277**	0.153
Large-stem fir	0.107	**−0.474**	**0.214**
*Rhododendron*	0.057	**−0.266**	**0.218**
Fruiting *Sorbus*	−0.036	−0.061	−0.004
Snag trees	0.027	**−0.259**	0.125
Logs	0.060	**−0.400**	**0.219**
Bamboo cover	**−0.514**	−0.062	0.100
Bamboo height	**−0.827**	−0.048	**0.172**
Canopy cover	−0.010	−0.113	0.020
*Total variation (%)*	*46.5*	*13.9*	*11.8*
*Cumulative variation (%)*	*46.5*	*60.4*	*72.2*

1 Values in **bold** are significant at P<0.05.

**Figure 5:**
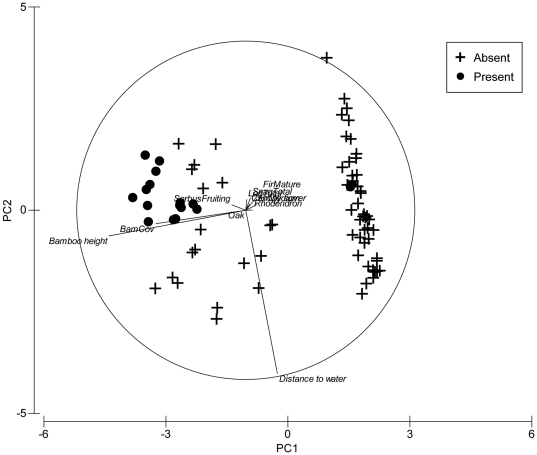
Principle Component Analysis showing relationship between ‘animal presence’ (plots with red panda evidence) and ‘animal absence’ plots in conifer forest, in Jigme Dorji and Thrumshingla National Parks, Bhutan.

A PCA within habitats reduced habitat variables into three principle components that explained 72.3% of the total variance in conifer forest and 73.1% in cool broadleaf forest. In conifer forest, 45.2% of the total variation was attributed to decreasing bamboo cover and height along PC1 (Table 4, Fig. 5). PC2 explained 15.9% of the total variation with distance to water increasing along the axis (Table 4, Fig. 5). PC3 explained 11.1% of total variation with mature fir, mid-stem fir, rhododendron, tree stump and snags, and fallen logs all decreasing along the axis (Table 4, Fig. 5).

**Table 4:** Principle Component scores^1^ for PCA of conifer forest plots in Jigme Dorji and Thrumshingla National Parks, Bhutan.

Variable	PC1	PC2	PC3
Altitude	0.025	−0.025	−0.045
Slope	−0.003	0.013	−0.029
Distance to settlement	0.028	−0.055	−0.042
Distance to road	−0.002	−0.060	**0.282**
Distance to water	0.186	**0.964**	−0.090
Large-stem trees	−0.023	0.058	−0.036
Mid-stem trees	0.031	−0.036	−0.150
Shrubs	0.015	0.072	−0.016
Mid-stem fir	0.065	−0.027	**−0.347**
Large-stem fir	0.095	−0.109	**−0.495**
*Rhododendron*	0.041	−0.001	**−0.308**
Fruiting *Sorbus*	−0.083	−0.034	0.078
Snag trees	0.028	−0.070	**−0.298**
Logs	0.007	−0.057	**−0.523**
Bamboo cover	**−0.532**	0.078	−0.045
Bamboo height	**−0.810**	0.150	−0.130
Canopy cover	0.025	−0.053	−0.190
*Total variation (%)*	*45.2*	*15.9*	*11.1*
*Cumulative variation (%)*	*45.2*	*61.1*	*72.3*

1 Values in **bold** are significant at P<0.05.

**Figure 6:**
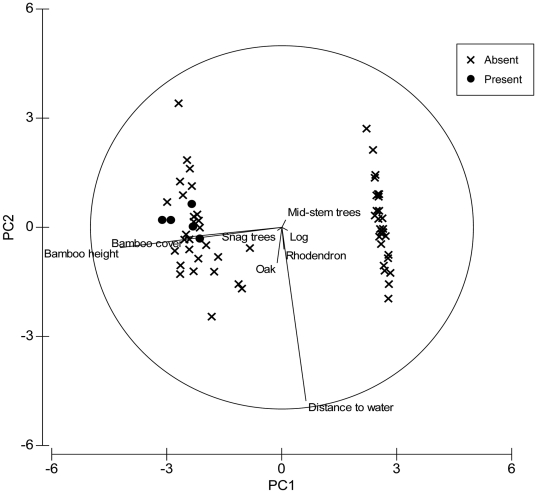
Principle Component Analysis showing relationship between ‘animal presence’ (plots with red panda evidence) and ‘animal absence’ plots in cool broadleaf forest, in Jigme Dorji and Thrumshingla National Parks, Bhutan.

PC1 in cool broadleaf forest explained 56.4% of the total variation with bamboo height and cover negatively associated with the axis (Table 5, Fig. 6). The distance to water was positively associated with PC2 and explained 11.0% of the total variance (Table 5, Fig. 6). PC3 explained 5.6% of the total variation with density of shrubs and number of oak trees decreasing along the axis, corresponding to an increase in distance to the nearest settlement, number of rhododendrons, tree stumps and snags, logs and canopy cover (Table 5, Fig. 6).

**Table 5:** Principle Component scores^1^ for PCA of cool broadleaf forest plots in Jigme Dorji and Thrumshingla National Parks, Bhutan.

Variable	PC1	PC2	PC3
Altitude	−0.030	0.014	−0.016
Slope	−0.032	0.037	−0.009
Distance to nearest settlement	−0.044	0.008	**0.283**
Distance to nearest road	0.043	0.013	−0.115
Distance to water	0.126	**0.968**	−0.004
Large-stem trees	0.012	0.092	0.116
Mid-stem trees	0.020	−0.059	−0.206
Shrubs	0.068	0.087	**−0.271**
Oak trees	0.019	−0.011	**−0.304**
*Castanopsis*	−0.001	0.034	−0.181
*Rhododendron*	0.011	0.121	**0.263**
Fruiting trees	−0.015	0.001	0.055
Tree stump and snag	−0.025	0.021	**0.473**
Log	0.029	0.031	**0.388**
Bamboo cover	**−0.506**	0.063	−0.021
Bamboo height	**−0.843**	0.120	−0.068
Canopy percent	−0.057	−0.083	**0.448**
*Total variation (%)*	*56.4*	*11.0*	*5.6*
*Cumulative variation (%)*	*56.4*	*67.5*	*73.1*

1 Values in **bold** are significant at P<0.05.

## Discussion

### Broad Habitat and Landscape Affiliations

Pradhan, Saha, and Khan recorded red pandas in cool broadleaf and conifer forests between 2800–3600 m asl at Singahalila National Park, India, and noted the importance of *Arundanaria* bamboo species [15]. Yonzon and Hunter recorded an affinity by red pandas for fir-*jhapra* bamboo forest between 2800–3900 m asl in Langtang National Park, Nepal [13], while two other studies suggested that red pandas were more common in East Himalayan Fir (*Abies spectabilis*) habitat in Kangchenjunga, Nepal [19], [21].

Red pandas in Jigme Dorji and Thrumshingla broadly occurred in cool broadleaf and conifer forests between 2110–4389 m asl, within Choudhury's predicted range of 1500–4800 m asl for red pandas in the Himalayan temperate forests [8]. However, our study firmly establishes the importance of Bhutan Fir (*Abies densa*) forests as the primary habitat for red pandas in Bhutan. Although present in spruce *Spicea spinulosa* and hemlock *Tsuga dumosa* dominated coniferous forests in Thrumshingla, red pandas showed a strong affinity for *Abies densa* forest between 2400–3700 m asl with a bamboo undergrowth of *Yushania* and *Arundanaria* spp. In the only other study of red pandas in Bhutan, Dorjee also recorded red pandas to occur predominantly in *Abies densa* forests between 2800–3700 m asl at Sakteng Wildlife Sanctuary, eastern Bhutan [22].

Despite the obvious importance of Bhutan Fir, red pandas were also recorded in cool broadleaf forest in both parks, albeit in low numbers of plots, suggesting that this habitat was used infrequently, or that they occurred at low densities in this forest type. Despite these low detection rates, this habitat may nevertheless be important to red pandas for connectivity between areas of prime habitat, during particular seasons, or for certain aspects of the species' ecology. More detailed investigation of red panda ecology and behaviour in cool broadleaf forest is therefore warranted.

Red pandas were detected more frequently on southerly and easterly slopes in our study, which supports similar observations by Reid, Hu and Yan who hypothesised that red pandas rested in direct sunlight during winter to reduce heat loss [9]. In Langtang National Park, Nepal, red pandas were not associated strongly with southerly slopes because longer sunlight periods there are not conducive to the growth of fir-*jhapra* bamboo forest [23]. We, however, noted that southerly and easterly slopes in our study areas had relatively high bamboo densities, which may be associated with sunlight and rainfall. Due to lack of detailed knowledge on bamboo ecology in Bhutan [24], more studies are needed to investigate the effects of physical landscape variables on bamboo.

### Fine-scale Habitat Correlates

When considered with a number of unrelated studies of broad habitat affiliations [11], [12], [13], [15], [21], [22] across the range of the species, our study confirms old growth fir (*Abies* spp. forest as the primary habitat for red pandas in the eastern Himalayas. Our multivariate analyses within mature temperate forest revealed the significance of a dense cover of *Yushania*, and *Arundanaria* bamboo spp.; a high density of fallen logs and tree stumps at ground level; a high density of trees, dead snags, and rhododendron shrubs in the mid-storey; and locations that were close to water. Red pandas probably rely on mature trees especially mature fir in conifer forest for resting, denning and escape from predators [13], [14]. High bamboo height and cover is possibly associated with its principal diet of bamboo leaves [10], [17] from common *Yushania* and *Arundanaria* bamboo spp. being readily accessed from mature trees, along with concealment from predators [8], [9], [13], [15]. Other important habitat elements like presence of mid-stem trees and rhododendron shrubs, fallen logs, tree stumps, and snags could be associated with suitable substrate for defecation [25] and for providing structural access to bamboo leaves [9], [10], [26]. Proximity to a water source is probably important to supplement the low water content associated with bamboo leaves [9], [13], [15], and was a strong predictor of red panda occurrence in our study.

Rowan (*Sorbus*) berries are probably an important dietary supplement for red pandas [7], since we detected red pandas around a variety of fruiting *Sorbus* trees (*S. microphylla*, *S. cuspidate*, *S. rufupilosa*, *S. lanata*, and *S. foliolosa*). Fruit, which has a higher caloric density than bamboo, may be a necessary supplement for lactating females [9]. This supplement may facilitate altitudinal movement by red pandas because such movements are largely dictated by the availability of seasonal *Sorbus* and *Rubus* berries [13], [27], [28].

### Anthropogenic Factors

Red pandas were not detected in forests close to settlements that were either dominated by oak such as *Q. Griffithii* and *Q. Semecarpifolia*, had sparse vegetation cover or had open areas with a high density of *Artemisia* shrubs. Oak forests in Bhutan are subject to high human pressure for pasture, litter collection and harvesting of oak, which is considered superior for firewood and agricultural tools [29]. Harvest of native timber is integral to the built environment in Bhutan, and demand is increasing from a growing population with higher living standards. A government policy of retaining traditional architecture is negatively impacting upon Bhutan's temperate forests, with 65% of Bhutan's households in rural areas exploiting potential red panda habitat to meet their needs, in conjunction with an equal urban-based demand for timber and firewood [7]. Open, *Artemisia*-dominated shrub areas close to settlements arise predominantly from ‘*Tsheri*’ (slash and burn agriculture), collection of firewood, and livestock grazing [30], [31]. Lack of cover in *Artemisia*-dominated shrubland probably provides inadequate food and shelter to red pandas, since we never detected red pandas in this modified habitat.

Despite their general reliance on bamboo, red pandas did not use the abundant dwarf *Yushania microphylla* bamboo in the open areas of the conifer forest whose growth is facilitated by browsing livestock and burning. Although red pandas and livestock are not thought to compete directly for food [13], grazing controls bamboo height [32], [33], which we determined to be an important habitat requirement for red pandas. The low quality dwarf bamboo would also require red pandas to spend longer periods foraging, making them more vulnerable to predators such as leopards (*Panthera pardus*) [13] and the ubiquitous domestic dogs belonging to herders and road workers [7]. Dwarf bamboo also remains under snow cover, making leaves inaccessible to red pandas during winter.

Bamboo harvesting, which includes collection of the species utilised by red pandas, constitutes a high-level threat to the species [34], in addition to intense grazing of bamboo by migratory herds of cattle and yaks [35]. Almost 42% of households in Bhutan use bamboo for roofing, thatching, fencing, baskets, arrows, containers, and other handicraft [36]. Apart from its socio-economic and ecological benefits to humans and red pandas, respectively, bamboo also assists in water conservation, land rehabilitation, carbon sequestration, and controlling soil erosion [37]. Excessive grazing in conifer forest can further reduce the regeneration of seedlings particularly of fir, hemlock and juniper [29], [38], which are important to red pandas.

### Conclusions

Our study firmly suggests that red pandas are primarily reliant on old-growth temperate fir forest with abundant bamboo undergrowth and readily available water sources. Because these forests in Bhutan are also integral to human subsistence and socio-economic development through collection of timber and non-timber forest products, our study now reveals a previously unrecognised and inadvertent conflict between the needs of people, and the needs of red pandas. However, because of the common reliance on forests by humans and red pandas, sustainable management of temperate forests to meet the needs of people will have a positive flow-on effect for red panda conservation, and generally contribute to Bhutan's philosophy of Gross National Happiness though the promotion of environmental conservation.

## Materials and Methods

### Ethics Statement

Interviews of park residents were conducted under University of New England's (UNE) Human Research Ethics Committee Approval HE10/078. Additional written consent to undertake the interviews in each national park was obtained from local government authorities (reference: JDNP/ADM-25/2010/124). Informed consent varied according to the respondents' literacy level; for respondents with minimal or no literacy (e.g. nomadic yak herders, road workers, farmers), we obtained their verbal consent in accordance with UNE's ethics committee guidelines for interviews involving respondents identified as being unable to read and write. We obtained written consent from literate respondents (e.g. government employees, park officials, school children), or in the case of minors, written consent was obtained from their guardians. For school children, additional written consent was also obtained from the local school board (reference: JDNP/ADM-25/2010/125) and from the school principal, before children were given the questionnaire to complete in class. All participation in our questionnaire survey was voluntary, and all respondents were treated as anonymous for the purposes of data analysis and manipulation.

### Study areas

Our study was conducted in Jigme Dorji National Park (‘Jigme Dorji’) (27°35′N to 28°12′N; 89°16′E to 90°17′E) and Thrumshingla National Park (‘Thrumshingla’) (27°12′N to 27°32′N; 90°44′E to 91°12′E) in Bhutan (Fig. 1) under permit number DF/Nga-4/08/1395 issued by the Department of Forests and Park Services, Bhutan. Jigme Dorji is mountainous and rugged (1400–7000 m asl), covering an area of 4349 km^2^. It abuts the Bhutan-China (Tibet) border to the north and west, while the southern and eastern boundary traverses the districts of Gasa, Thimphu, Paro, Punakha and Wangdiphodrang. The vegetation in the park can be categorized into five main habitat types: warm broadleaf forest (1400–2000 m asl; common species include *Macaranga postulata*, Glaucous-leaf Oak *Quercus glauca*, *Toricellia tiliifolia*, *Mussaenda roxburghii*, *Schima wallichii*, *Ficus roxburghii* and *Engelhardia spicata*); chirpine forest (1400–1800 m asl) on dry south facing slopes (common species include Chirpine *Pinus roxburghii*, *Woodfordia fruticosa*, *Buddleja bhutanica*, and the Tree Rhododendron *Rhododendron arboreum*); temperate cool broadleaf forest (hereafter called cool broadleaf forest; 2200–3000 m asl; common species include Brown Oak *Q. semecarpifolia*, *Q. griffithii*, *Symplocos dryophila*, Campbell's Maple *Acer campbellii*, Alder-leaf Birch *Betula alnoides*, and Himalayan Hornbeam *Carpinus viminea*); temperate conifer forest (hereafter called conifer forest; 2000–4000 m asl; common species include Blue Pine *P. wallichiana*, Himalayan Hemlock *Tsuga dumosa*, Sikkim Spruce *Picea spinulosa*, Bhutan Fir *Abies densa*, Rowan *Sorbus* spp., *Rhododendron* spp., and bamboo species such *Yushania microphylla*, *Y. maling*, *Y. hirsuta* and *Arundanaria maling*); and alpine meadows (4000–5000 m asl). The average annual precipitation in Gasa (elevation: 2760 m asl, Fig. 1) is 2250 mm with mean annual temperatures ranging from 6°C to 16°C. Average annual precipitation in Taashingthang (elevation: 1600 m, Fig. 1) is 1500 mm and mean annual temperatures range from 12°C to 20°C. Rainfall mostly occurs between June and August [39].

Jigme Dorji is an important regional biodiversity reserve. It harbours more than 1500 plant species from 144 families, over 300 species of birds, and over 40 species of mammals including high priority species like the tiger *Panthera tigris*, snow leopard *Uncia uncia*, red panda, takin *Budorcas taxicolor*, and Himalayan black bear *Ursus thibetanus*. There are approximately 6,500 human residents, mainly nomadic yak herders, who are dependent on the park resources for their livelihood [40].

Thrumshingla is located in Central Bhutan, covering an area of 905 km^2^ within the districts of Bumthang, Mongar, Zhemgang and Lhuntse (Fig. 1). Thrumshingla has a rugged topography with an elevation range of 650–4500 m asl. Five major habitat types are present: subtropical forest (150–1000 m asl; common species include Cotton Tree *Bombax ceiba*, Duabanga *Duabanga grandiflora*, Agarwood *Aquilaria agallocha*, and silktrees *Albezia spp.*); warm broadleaf forest (1000–2000 m asl.; common species include *Altingia excelsa*, *Castanopsis indica*, *Engelhardia spicata*, Mallato *Macaranga postulata*, *Schima wallichii*, *Stereospermum personatum*, and figs *Ficus* spp.); cool broadleaf (2000–2900 m asl.; common species include oak, *Quercus* spp. *Castanopsis hystrix*, birch *Betula* spp., maple *Acer* spp., *Symplocos dryophila*, Campbell's Magnolia *Magnolia campbellii*, Globe Magnolia *M. globossa*, and Common walnut *Juglans regia*); conifer forest (2500–4000 m asl; common species include Blue Pine, Bhutan Fir, Himalayan Hemlock, Sikkim Spruce, juniper *Juniperus* spp., and *Rhododendron spp.*); and alpine meadows (above 4000 m asl).

Average annual rainfall in Thrumshingla varies from 700–1500 mm and mainly occurs between June and August. The mean annual temperature in the higher elevated northern parts of the park ranges from 3°C–15°C, whereas at lower elevations (at Lingmethang, 650 m asl) temperatures range from 17°C–29°C [39].

More than 1000 species of plants, 360 species of birds, and 69 species of mammals have been recorded in Thrumshingla (Thrumshingla National Park 2008). Priority mammal species present include tiger, red panda, Himalayan black bear, goral *Nemorhaedus goral*, Himalayan musk deer *Moschus chrysogaster*, and capped langur *Trachypithecus pileatus* [41]. Approximately 5600 people from 1165 households live within or on the boundary of the park and are largely dependent on park resources for their livelihoods [41].

### Presence-absence surveys

Red panda populations occur at different elevation ranges and in different forest types across the eastern Himalayas in China, India and Nepal [8]; data on broad distribution of red pandas in Bhutan is limited to only anecdotal observations and preliminary surveys [7], [22]. From November 2007 to April 2008 we established 339 random 20 m×20 m plots along 339 km of transects (n = 6, range = 21–84 km, mean = 33 km) that traversed the five major habitats in Thrumshingla (Fig. 1). Human trails and cattle migratory routes were utilised because of difficulties in establishing straight line transects in steep terrain. Transects were carefully selected to ensure adequate coverage of all habitats within the altitudinal range of the park. Plots were established at 1 km intervals along each transect, with the centre of each plot located 150-m away along a random direction (0°–360°). We recorded all evidence (droppings, spoor, vocalizations, and direct sightings) of wildlife within each plot. Red panda presence was confirmed through their droppings or through direct sightings of an individual. To obtain additional location data on red pandas in Thrumshingla, we interviewed 324 park residents comprising farmers, cattle herders, students, teachers, road workers, monks, civil servants as well as park and forestry officials. Similar interviews (n = 340) were conducted in Jigme Dorji to assess locality records for follow up site selection to evaluate microhabitat association.

### Microhabitat association

From December 2007 to December 2009, we carried out systematic stratified sampling along 46 survey transects (n = 46, range = 2–3 km, mean = 2.5 km), 22 transects in Jigme Dorji (total length = 67 km) and 24 transects in Thrumshingla (total length = 71 km). We sampled all habitat types in proportion to their availability (Fig. 1).

We established ‘animal-presence’ and ‘animal-absence’ plots, using droppings as an indicator of red panda presence, in line with several other studies [15], [17], [42]. At 500-m intervals along each transect, a point 150 m away was selected along a random direction (0–360°) and scanned for red panda droppings within a 50-m radius. If droppings were found, we established a 20 m×20 m plot centred on the pellet group and measured a range of habitat variables (Table 1). If no red panda droppings were found, we measured similar habitat variables within a 20 m×20 m plot centred on the 150 m point for comparison with animal-presence plots. Within both animal-presence and animal-absence plots, we further established a 5 m×5 m sub-plot around the centre to quantify the density of bamboo, bamboo height and number of shrubs (height: <1 m). We established additional animal-presence plots in both parks when red pandas were opportunistically sighted or their droppings discovered during fieldwork, using the location of the animal or droppings as the centre of the plot. Plots were monitored seasonally between December 2007 and December 2009.

### Data Analysis

Where statistically justified [43], data were pooled by park, season and habitat types and analyzed with SPSS 18 (IBM Corporation, USA; www-01.ibm.com/software/), R 2.10.1 (R Foundation for Statistical Computing, www.r-project.org/), PRIMER 6 (Plymouth Routines in Multivariate Ecological Research, UK; www.primer-e.com/primer.htm), PRESENCE 2.3 (Patuxent Wildlife Research Center, USA; www.mbr-pwrc.usgs.gov/software/presence.html) and ArcGIS 9.3 (ESRI, USA; www.esri.com/software/arcgis/). We used non-parametric procedures [44] in all statistical testing.

Diversity of tree and shrub species in a plot was determined by a Simpson's diversity index [45]. An Important Value Index (IVI) of tree species in each plot was determined by summing relative frequency, relative abundance and relative dominance [46]. Following Clarke [47], a Principle Component Analysis (PCA) was performed on habitat variables (Table 2). Tree species with an IVI of ≥10 were also incorporated into the PCA. We included all *Sorbus* spp trees in the PCA because *Sorbus* fruit is a dietary supplement for red pandas [7]. Before a PCA, all habitat variables were fourth-root transformed to prevent highly abundant species from influencing the similarity measure [47]. We utilized a Bray-Curtis similarity for all subsequent PCAs'. Red panda presence-absence plots and associated forest types were treated as fixed factors. To investigate general trends in habitat use, we conducted an initial PCA on pooled data from habitats confirmed to have red pandas. We then conducted separate PCAs' on each habitat to look for habitat-specific trends.
